# The relative importance of abiotic and biotic environmental conditions for taxonomic, phylogenetic, and functional diversity of spiders across spatial scales

**DOI:** 10.1007/s00442-023-05383-0

**Published:** 2023-06-01

**Authors:** Johannes Måsviken, Love Dalén, Karin Norén, Fredrik Dalerum

**Affiliations:** 1 Department of Zoology, Stockholm University, Stockholm, Sweden; 2 Department of Bioinformatics and Genetics, Swedish Museum of Natural History, Stockholm, Sweden; 3 Centre for Palaeogenetics, Stockholm, Sweden; 4 Biodiversity Research Institute (University of Oviedo-Principality of Asturias-CSIC), Spanish National Research Council, Research Building, Mieres Campus, 33600 Mieres, Spain; 5 Department of Zoology and Entomology, Mammal Research Institute, University of Pretoria, Hatfield, South Africa

**Keywords:** Elevation, Environmental gradients, Biodiversity dimensions, Taxonomic diversity, Phylogenetic diversity, Functional diversity, Community regulation, Spatial scale, Araneae

## Abstract

Both abiotic and biotic conditions may be important for biodiversity. However, their relative importance may vary among different diversity dimensions as well as across spatial scales. Spiders (Araneae) offer an ecologically relevant system for evaluating variation in the relative strength abiotic and biotic biodiversity regulation. We quantified the relative importance of abiotic and biotic conditions for three diversity dimensions of spider communities quantified across two spatial scales. Spiders were surveyed along elevation gradients in northern Sweden. We focused our analysis on geomorphological and climatic conditions as well as vegetation characteristics, and quantified the relative importance of these conditions for the taxonomic, phylogenetic, and functional diversity of spider communities sampled across one intermediate (500 m) and one local (25 m) scale. There were stronger relationships among diversity dimensions at the local than the intermediate scale. There were also variation in the relative influence of abiotic and biotic conditions among diversity dimensions, but this variation was not consistent across spatial scales. Across both spatial scales, vegetation was related to all diversity dimensions whereas climate was important for phylogenetic and functional diversity. Our study does not fully support stronger abiotic regulation at coarser scales, and conversely stronger abiotic regulation at more local scales. Instead, our results indicate that community assembly is shaped by interactions between abiotic constrains in species distributions and biotic conditions, and that such interactions may be both scale and context dependent.

**Supplementary Information:**

The online version contains supplementary material available at 10.1007/s00442-023-05383-0.

## Introduction

Biodiversity can be critically important for ecosystem function and stability as well as for important ecosystem services (Hooper et al. 2005; Balvanera et al. 2006; Cardinale et al. 2012). Therefore, quantifying how biodiversity is regulated and maintained has been a central quest in modern ecology (e.g., Rosenzweig 1995; Chesson 2000; Hubbell 2001), which urgency has been accentuated by an accelerating global change (Lovejoy and Hannah 2019). While abiotic conditions, i.e., non-living components of the environment such as light, climate, and geomorphology, can have profound effects on biodiversity by setting the abiotic boundaries for species existence (Körner and Paulsen 2004; Clarke et al. 2013), interactions among species such as competition, predation, and facilitative interactions may also affect biodiversity by influencing local abundances and species compositions (Chapin et al. 1997; Wisz et al. 2013). Hence, abiotic and biotic environmental conditions interact in regulating biodiversity, but relative effect is expected to vary predictably with spatial scales (Menge and Olson 1990). Abiotic characteristics are often thought of as ‘environmental filters’, which generally are regarded to restrict species pools across large spatial scales (Kraft et al. 2015). Biotic regulation, on the other hand, relies on direct species interactions which requires that species have the opportunity to interact (Weiher et al. 2011). Therefore, there is an expected shift in community regulation from abiotic regulation at large spatial scales toward an increasing importance of biotic regulation at more local scales.

Biodiversity is most often measured as taxonomic richness or through a variety of indices that weigh taxonomic richness by relative abundances (Magurran 2004). However, species are generally not equally different, neither in their evolutionary histories nor in their phenotypic characteristics (Vellend et al. 2011; Weiher et al. 2011). To account for variation which is not captured by taxonomy alone, diversity can also be quantified as phylogenetic and functional diversity, where the former directly measures phylogenetic variation within species communities and the latter phenotypic variation (Faith 1992; Tilman 2001). While taxonomic, phylogenetic, and functional diversity obviously are related for any given species community, their relationships depend on the evolutionary history of the taxa as well as on the phylogenetic signal in expressed phenotypes (Blomberg et al. 2003). In species communities where all taxa are equally evolutionary distant, and where phenotypes are completely phylogenetically linked, all three dimensions will be perfectly correlated. This is, however, rarely the case in ecological communities. Therefore, the interpretation of taxonomic diversity is dependent on the variation in phylogenetic relatedness among taxa as well as in their phenotypic variation (Leinster and Cobbold 2012). Phylogenetic and functional diversity, on the other hand, directly reflect different properties of communities, where functional diversity is directly related to contemporary ecosystem performance and resilience (Tilman et al. 2001), whereas phylogenetic diversity is related to future ecosystem stability (Dalerum 2013).

Elevational gradients are frequently used as proxies for environmental characteristics, since they offer broad variations in climate and productivity over relatively limited geographic distances (Lomolino 2001). Although variation in taxonomic diversity is well documented along elevational gradients (e.g., Terborgh 1977; Rahbek 1995; McCain 2005), there is no consensus regarding the underlying mechanisms driving such variation (Willig et al. 2003; Currie et al. 2004). Multiple mechanisms have been suggested, such as the reduction in available area, climate, net available energy, and evolutionary effects of shifting environmental conditions (Wright 1983; Rosenzweig 1995; Allen et al. 2002; Rahbek et al. 2019a, b; Tietje et al. 2022). However, few studies have explicitly addressed how local environmental conditions influence biodiversity surveyed along elevational gradients, but rather used the gradients as a proxy for assumed environmental variation (Körner 2007). This is unfortunate, since direct quantifications of the effects of environmental conditions could generate a more comprehensive mechanistic understanding of how biodiversity is regulated.

Spiders (Araneae) offer useful model systems for evaluating how the relative effects of abiotic and biotic conditions vary across diversity dimensions and spatial scales. Spiders are among the most widely distributed and numerous arthropods on Earth (Turnbull 1973), and are easily sampled. Spider diversity vary along primary productivity gradients (Whitehouse et al. 2009; Piel 2018), and multiple environmental conditions may regulate the composition of local spider assemblages (Jiménez-Valverde and Lobo 2007; Ernst et al. 2016). They have, therefore, been suggested as an informative organism group for biodiversity monitoring (Bowden and Buddle 2010), particularly in environments at high latitude and elevation (Hodkinson 2005; Gillespie et al. 2019). Spiders are generalist predators that feed on a wide variety of arthropods, primarily insects as well as other arachnids (Nentwig 1987), and are important for ecosystem functioning and stability (Schmitz 2003).

In this study, we quantify the relative importance of abiotic and biotic conditions for taxonomic, phylogenetic, and functional diversity of spider communities surveyed along elevation gradients in the Swedish mountains. We evaluate possible effects across two spatial scales, one intermediate reflecting approximately 500 m and one local reflecting communities within 25 m, and focus our analysis on geomorphological and climatic conditions as well as on vegetation characteristics. We explicitly test the following two predictions: (I) abiotic conditions will be more important at the intermediate than at the local spatial scale and biotic conditions more important at the local scale; (II) abiotic conditions will be more important for phylogenetic than for functional diversity, and biotic conditions will me more important for functional than for phylogenetic diversity. We base these predictions on the predictable scale dependence in the relative strength of abiotic and biotic community regulation, on the assumption that phylogenetic diversity reflects evolutionary adaptations to the abiotic environment, and on the assumption that functional diversity, i.e., phenotypic variation, will be regulated by species interactions (Weiher et al. 2011).

## Methods

### Study area

**Fig. 1:**
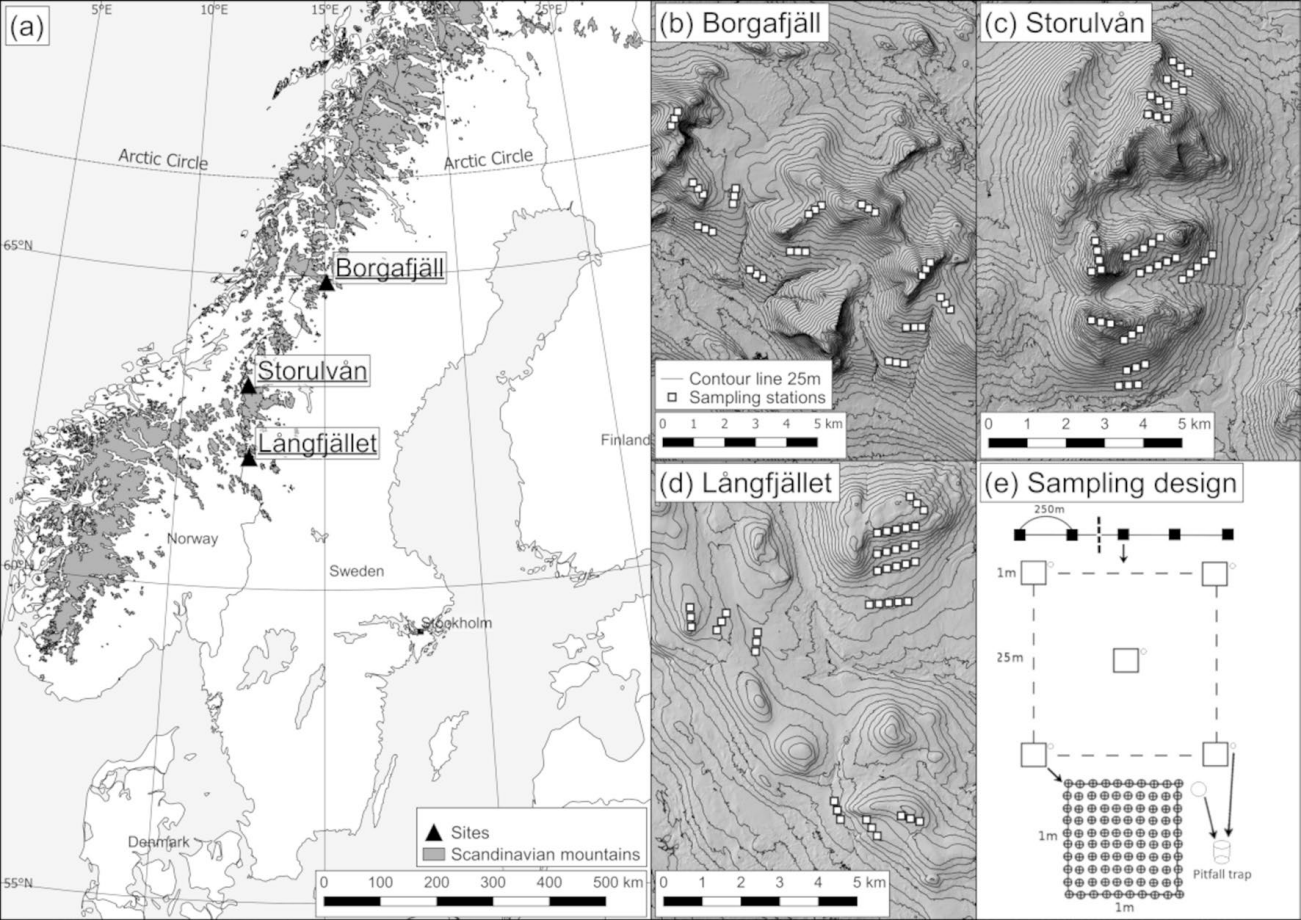
The locations of the study sites within the Swedish mountains (**a**), as well as detailed maps of the distribution of the sampling stations within each site (**b–d**). Within each site, sampling was done along transects consisting of three or five sampling stations spaced 250 m apart, each station in turn consisting of five 1 m^2^ sampling plots, each with one pitfall trap (**e**). Within each plot, vegetation cover and plant species were recorded and 25 intercept points in a 10 × 10 cm grid were used for relative abundance and vegetation height measurements

The study was conducted above the tree line on oroarctic tundra at three locations in the Swedish part of the Scandinavian Mountains (hereafter referred to as the “Swedish mountains”). The Scandinavian Mountains extend for approximately 1500 km from the southern part of Norway towards northeast, along the border with Sweden up to the arctic coast (Fig. 1). The tree line, which globally occurs at ground temperatures of ~ 7 °C (Körner and Paulsen 2004), varies between approximately 600–1000 m above sea level (m.a.s.l.) in Sweden (Odland 2015). The tree line is primarily formed by mountain birch (*Betula pubescens* subsp. *czerepanovii*). Maximum elevation of the Scandinavian mountains is 2469 m.a.s.l. However, despite the relatively modest elevations, the climate is equivalent to higher mountain ranges due to the high latitude, with minimum monthly average air temperatures of approximately − 8 °C during the winter and 9 °C during the summer. Monthly average precipitation is generally lower during winter (~ 64 mm) than summer (~ 95 mm). The vegetation above the tree line is dominated by oroarctic heath consisting of dwarf shrubs, for instance *Empetrum nigrum* subsp. *hermaphroditum*, *Salix* spp., *Vaccinium* spp., as well as graminoids including species of Poaceae, *Carex*, and *Juncus* (Måsviken et al. 2020). Wet areas such as bogs are dominated by sedges, and grasses such as *Carex spp.*, *Eriophorum* spp. and *Nardus stricta*, as well as magnoliopsids like *Andromeda polifolia* and *Rubus chamaemorus* (Carlsson et al. 1999).

There have been 740 spider species recorded as reproducing in Sweden, with Linyphiidae (310 species), Theridiidae (60 species), and Lycosidae (58 species) being the most species rich families (Dyntaxa 2022). However, there is currently no data on the numbers of species found in the Swedish mountain areas.

### Spider collection and taxonomic identification

We conducted sampling at three locations ranging in latitude from 64.90° to 62.10°; Borgafjäll, Storulvån and Långfjället (Fig. 1a). Topography at all sites consists of higher peaks surrounded by lower undulating valleys. Sampling at Borgafjäll was conducted in an area of 32 km^2^, Storulvån in an area of 16.3 km^2^ and at Långfjället in an area of 30.8 km^2^. Sampling elevations ranged from 840 to 1435 m.a.s.l. (Table 1).

**Table 1:** Characteristics of the study sites including geographic coordinates, elevation of highest peak, elevation of closest tree line, sampled elevation range, bedrock characteristics (SGU 2021), average monthly temperature (Meineri and Hylander 2017), and precipitation (Fick and Hijmans 2017) for the summer and winter months

	Borgafjäll	Storulvån	Långfjället
Coordinates	lat 64.90° lon 15.04°	lat 63.21° lon 12.34°	lat 62.10° lon 12.43°
Highest peak in massif	1477 m.a.s.l.	1463 m.a.s.l.	1204 m.a.s.l.
Elevation of closest tree line	~ 775 m.a.s.l.	~ 800 m.a.s.l.	~ 840 m.a.s.l.
Sampled elevation range	~ 840–1435 m.a.s.l.	~ 840–1410 m.a.s.l.	~ 875–1170 m.a.s.l.
Bedrock characteristics	Amphibolite, mica rich slate, ultramafic intrusive	Paragneiss, mica rich slate, amphibolite	Arkose, quartz arenite
*Average monthly temperature*			
Summer	8.0 °C	8.4 °C	9.7 °C
Winter	− 8.2 °C	− 6.8 °C	− 8.3 °C
*Average monthly precipitation*			
Summer	98 mm	97 mm	91 mm
Winter	78 mm	67 mm	48 mm

At each site, spiders were collected using a stratified random sampling design using pitfall traps placed along transects of either 500 m (twelve in Borgafjäll, eight in Storulvån, seven in Långfjället) or 1000 m (four at both Storulvån and Långfjället). Transects were stratified according to elevation at each site by dividing the elevation extent from the tree line to the highest peak into four elevation bands (Fig. 1b–d). The transects consisted of sampling stations spaced 250 m apart, three stations for the 500 m transects, and five stations for the 1000 m long transects. Each station consisted of five pitfall traps placed in a 25 m square with one trap in each corner and one in the center (Fig. 1e). The pitfall traps were filled with water and a mild unscented detergent to break surface tension. The sampling was done during mid-July to early August at all sites, during 2018 for Långfjället and Storulvån and 2019 for Borgafjäll. The traps were left out for 5 days before collection, corresponding to roughly 10–20% of the summer period in these mountain areas. All collected spider specimens were put in separate tubes and stored in 95% ethanol at − 20 °C until further identification.

Taxonomic identification of spiders was done morphologically by taxonomic expertise to species or the closest possible taxonomic rank following the nomenclature in Dyntaxa (2022).

Each specimen was classed as either adult or juvenile based on size and general characteristics as well as genitalia if needed, and its total body length from the tip of the cephalothorax to the end of the abdomen was measured. To get representative size data, only adult spiders were used for quantifying diversity.

### Quantification of diversity dimensions

Quantification of biodiversity dimensions of spider communities was done for two geographic scales, one pooling all spiders collected within one transect (intermediate scale) and one pooling all spiders from within one sample station (local scale). For the intermediate scale, the 1000 m transects were subseted to three stations to make them comparable to the shorter 500 m transects. Subsetting was done by removing the last two stations at each transect. These two scales, hence, represent spider alpha diversity across 500 m (intermediate scale) or 25 × 25 m (local scale).

We quantified taxonomic diversity using the Shannon diversity index (Shannon 1948) calculated on proportional abundance from the number of collected spiders for every taxon in each transect or station. We quantified phylogenetic and functional diversity using a Shannon index calculated on abundance weighted species contributions to individual branches in phylogenetic trees or functional dendrograms (Allen et al. 2009). Hence, these metrics include information on the relative abundances of species, which was lacking from Faith's (1992) and Petchey and Gaston's (2002) definitions of branch length-based diversity. We calculated diversity metrics for each sample (individual transect or station) by pruning the full tree or dendrogram to contain only species occurring at that sampling unit. We based phylogenetic diversity quantification on a phylogeny generated from mitochondrial cytochrome c oxidase subunit 1 (COI) sequences (Supplementary information, Appendix 1, Figure S1) and functional diversity on a dendrogram constructed from a matrix of five traits related to body size, hunting mode, web type, diet, and dispersal characteristics (Supplementary information, Appendix 1, Table S1). For phylogenetic diversity, we opted to make our own tree in the lack of a larger consensus phylogeny including the taxa relevant for our study. For functional diversity, the trait values were either derived from our own measurements (body length) or compiled from the literature (all other traits). We compiled trait data from the literature to species rank if possible, and if not, we either used information for the most closely related species or the closest higher taxon. The dendrogram was constructed by first calculating a pairwise distance matrix using Gower distances (Gower 1971), which was clustered into a dendrogram using the unweighted average linkage clustering (UPGMA), since this method provided the highest cophenetic correlation (*r* = 0.89) (Supplementary information, Appendix 1, Figure S2).

### Environmental conditions

We used environmental conditions relating to site-specific characteristics in geomorphology, climate, and vegetation. We selected four uncorrelated characteristics (*r* < 0.8) for each of these groups. Geomorphological characteristics included aspect (direction of slope), slope steepness, topographic wetness index (TWI), and bedrock silica content. Climate characteristics included average annual temperature, monthly temperature variation, average monthly precipitation, and monthly precipitation variation. Vegetation characteristics included vascular plant diversity, vascular plant cover, moss cover, lichen cover, and maximum vascular plant height variation. Detailed descriptions of the calculations of environmental characteristics are given in Supplementary Information, Appendix 2.

### Data analysis

We used linear mixed effect models to evaluate pairwise relationships between the different diversity metrics for both spatial scales. We used taxonomic diversity as the predictor for the models including this metric and phylogenetic diversity as the predictor for the models relating phylogenetic to functional diversity. We highlight that the selected metrics for phylogenetic and functional diversity cannot be negatively related to taxonomic diversity, but that the strength of the effect of taxonomic richness is dependent on the phylogenetic similarity or phenotypic overlap among species within each assembly (Dalerum et al. 2012).

We used three heuristic methods to evaluate the relative importance of the three groups of environmental conditions on each spider diversity dimension across the two spatial scales. All three methods were based on information theoretic approaches (Burnham and Anderson 2004).

The first approach evaluated the relative importance of environmental conditions using model ranking based on Akaike's information criterion corrected for small sample sizes (AICc values, Akaike 1974). For this approach, we created six sets of linear mixed models, one set for each spatial scale and diversity dimension. Each model set contained a full model, including all environmental conditions as fixed predictors, as well as three models only including the four characteristics in each group of environmental conditions (i.e., geomorphology, climate, vegetation). In all models, respective spider diversity was used as the response variable. The models were ranked for each spatial scale and diversity dimension, where models within two AICc units were regarded to have had approximately equal empirical support (Burnham and Anderson 2004). For all models, we also calculated the marginal R^2^, i.e., the variance explained by the fixed terms following Edwards et al. (2008).

Our second approach evaluated the relative importance of individual environmental characteristics based on their frequency of occurrences in models selected from sets of linear mixed models containing all possible combinations of environmental characteristics. We created six full model sets, one set for each diversity dimension and spatial scale. Each of these 6 sets consisted of 4096 different models. From each of these sets, we selected all models within two AICc units of the model with the lowest AICc value (Δ AICc) and used the frequency of occurrences of each environmental characteristic in the selected models as a heuristic index of their relative importance.

Our third approach evaluated the relative strength of the effects of individual environmental characteristics based on AICc weighted model averaging of individual parameter estimates. For this approach, we used the same set of selected models as described above. For each selected model, we calculated the Akaike weight as the relative likelihood of the model divided by the sum of the relative likelihoods for all models in a model set. It can take a value between 0 and 1 (Burnham and Anderson 2004). The Akaike weights were calculated separately for each diversity metric and spatial scale. We then used the Akaike weights to calculate weighted averages for each of the parameter values. We averaged parameter values over all models even if a particular parameter was not included in a particular model. For models where a particular parameter was absent, we set its parameter value to 0 in the average calculations. All the parameters were scaled to unit variance to enable direct comparison among environmental characteristics and models.

For all models, we added site as a random term for the intermediate scale and transect nested in site for the local scale. Using this random effect structure, there were no detectable spatial autocorrelation in the residuals evaluated using the Moran’s *I* test (Supplementary information, Appendix 1, Table S2).

All analyses were performed in the R statistical environment (version 4.0.4, http://www.r-project.org) and the contributed packages vegan (version 2.5-7, Oksanen et al. 2022), dplyr (version 1.0.5, Wickham et al. 2021), ape (version 5.5, Paradis and Schliep 2019), lme4 (version 1.1-27, Bates et al. 2015), lmerTest (version 3.1-3, Kuznetsova et al. 2017), MuMIn (version 1.46.0, Barton 2020), r2glmm (version 0.1.2. Jaeger 2017), Spdep (1.2-3, Bivand and Wong 2018).

## Results

We collected a total of 1930 adult spiders, with a decreasing number of spiders collected per site going from north towards the south (Borgafjäll 705 spiders, Storulvån 696 spiders, and Långfjället 528 spiders). However, the sampling effort was not even, with 36 stations sampled at Borgafjäll, 42 at Storulvån, and 41 at Långfjället. There was also an unequal number of disturbed traps among the three sites. Hence, the collected numbers should not be interpreted as an index of local abundances. In total, we identified 62 spider taxa belonging to 8 different families; Clubionidae, Gnaphosidae, Hahniidae, Linyphiidae, Lycosidae, Philodromidae, Theridiidae, and Thomisidae. Of these taxa, 58 were determined to species, 3 to genus and 1 to family. Linyphiidae and Lycosidae were the most abundant as well as contained the most sampled taxa (Table 2). Spiders from Linyphiidae, Lycosidae, and Gnaphosidae occurred on all sites whereas spiders from Clubionidae, Hahniidae, Philodromidae, and Theridiidae only were sampled in very low numbers on a subset of the sites. We found 36 taxa at Borgafjäll, 43 at Storulvån, and 35 at Långfjället. For the intermediate scale, which were subsampled to only contain 3 stations per transect, we based our analyses on 57 taxa, of which 53 were identified to species, 3 to genus, and 1 to family. Of these, 36 taxa were found at Borgafjäll, 37 at Storulvån, and 32 at Långfjället (Supplementary information, Appendix 1, Table S3). The number of sampled spiders at each site appear to have captured the majority of available taxa (Supplementary information, Appendix 1, Figure S3).

**Table 2:** Number of identified taxa as well as the number of sampled individuals in the eight identified spider families, both for all sites pooled as well as for each of three sites sampled along the Swedish mountains

Family	All sites		Borgafjäll		Storulvån		Långfjället	
	Taxa	*n*	Taxa	*n*	Taxa	*n*	Taxa	*n*
Clubionidae	1	1					1	1
Gnaphosidae	4	121	2	16	3	38	4	67
Hahniidae	1	3			1	2	1	1
Linyphiidae	45	912	25	370	31	474	18	68
Lycosidae	10	858	6	316	7	176	8	366
Philodromidae	1	1					1	1
Theridiidae	2	2	1	1			1	1
Thomisidae	2	32	2	2	1	6	2	24

### Relationships between diversity dimensions

**Fig. 2:**
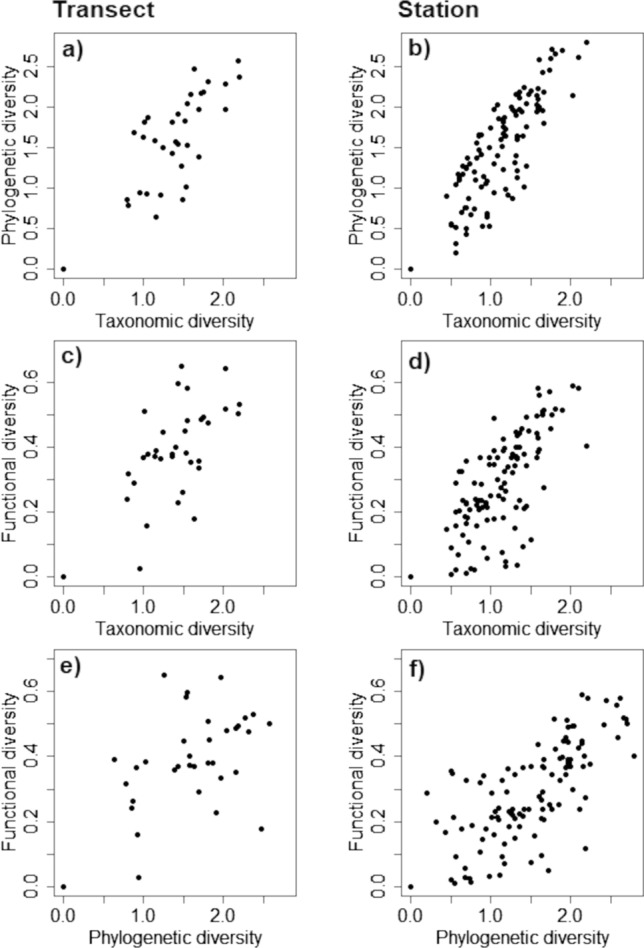
Relationships between taxonomic, phylogenetic, and functional dimensions of spider diversity. Each data point represents the diversity estimate based on the collected spiders pooled along a full transect (**a, c, e**) or within a single sample station (**b, d, f**) for three combinations of diversity dimensions: taxonomic and phylogenetic diversity for the intermediate (**a**) and the local scale (**b**), taxonomic and functional diversity for the intermediate (**c**) and the local scale (**d**), as well as phylogenetic and functional diversity for the intermediate (**e**) and the local scale (**f**)

Although all relationships between diversity dimensions were significantly positive, they were weaker at the intermediate than at the local scale, with the scale dependencies being particularly strong for the relationships between phylogenetic and functional diversity. For the intermediate scale, taxonomic diversity was positively related to phylogenetic diversity with an *R*^2^ = 0.56 (*F* = 43.58, *df* = 1,34, *p* < 0.001, Fig. 2a, whereas the relationship was much stronger at the local scale (*R*^2^ = 0.81, *F* = 513.40, *df* = 1,118, *p* < 0.001, Fig. 2b). Similar relationships were observed between taxonomic and functional diversity (intermediate scale: *R*^2^ = 0.41, *F* = 23.70, *df* = 1,34, *p* < 0.001, Fig. 2c; local scale: *R*^2^ = 0.63, *F* = 205.00, *df* = 1,118, *p* < 0.001, Fig. 2d), as well as between phylogenetic and functional diversity (intermediate scale: *R*^2^ = 0.26, *F* = 11.92, *df* = 1,34, *p* = 0.002, Fig. 2e; local scale: *R*^2^ = 0.63, *F* = 197.47, *df* = 1,118, *p* < 0.001, Fig. 2f).

#### Relative importance of environmental conditions

For spider taxonomic diversity, the models including vegetation were regarded as the most appropriate for both the intermediate and the local scale. In contrast, the most appropriate model for spider phylogenetic diversity at the intermediate scale included all groups of environmental conditions, whereas the most appropriate model for the local scale only included climate conditions. Similarly, both the model including all groups of environmental conditions as well as the one including only vegetation and the one with only climate conditions were regarded as the most appropriate for spider functional diversity at the intermediate scale, whereas only the model including vegetation was regarded as the most appropriate for the local scale. The explained variance of the fixed environmental conditions in these models was modest, but generally higher for the intermediate than for the local scale (Table 3).

**Table 3:** AICc-based model rank, values of Akaike's information criterion relative to the model with the lowest value (Δ AICc values), marginal *R*^2^ as well as their associated *p* values for candidate models for spider taxonomic, phylogenetic, and functional diversity for two spatial scales, intermediate and local

Environmental conditions	Taxonomic diversity				Phylogenetic diversity				Functional diversity			
	Rank	Δ AICc	*R*^2^	*p*	Rank	Δ AICc	*R*^2^	*p*	Rank	Δ AICc	*R*^2^	*p*
*Intermediate scale*												
All	2	2.08	0.63	< 0.001	1	0	0.76	< 0.001	1	1.33	0.55	< 0.001
Geomorphologic	4	13.02	0.19	0.130	4	22.00	0.28	0.020	2	4.74	0.21	0.090
Climatic	3	7.39	0.31	0.010	2	6.09	0.55	< 0.001	1	0	0.31	0.010
Vegetation	1	0	0.44	< 0.001	3	9.20	0.40	< 0.001	1	0.68	0.30	0.020
*Local scale*												
All	2	6.57	0.35	< 0.001	3	4.12	0.44	< 0.001	3	3.51	0.41	< 0.001
Geomorphologic	4	18.30	0.04	0.300	4	21.20	0.03	0.510	4	24.42	0.06	0.120
Climatic	3	8.85	0.21	< 0.001	1	0	0.37	< 0.001	2	3.29	0.32	< 0.001
Vegetation	1	0	0.29	< 0.001	2	3.65	0.32	< 0.001	1	0	0.39	< 0.001

For each model set (i.e., diversity metric and spatial scale), the candidate models contained either all or one of three groups of environmental conditions relating to geomorphology (aspect, slope, TWI, bedrock silica content), climate (annual average temp., temp. variation, monthly average precipitation, within year precipitation variation), and vegetation (vascular plant diversity, vascular plant cover, moss and lichen cover, maximum vascular height variation). Models within two AICc units of the model with the lowest AICc value have been given equal rank

### Relative importance of individual environmental characteristics

For spider taxonomic diversity, only 1 model was selected at the intermediate scale (Table 4) but 28 models at the local scale (Table 5, Supplementary information, Appendix 1, Table S5). The model at the intermediate scale included monthly average precipitation, vascular plant diversity and plant cover, whereas the models at the local scales included predictors from all groups of environmental conditions, but with vascular plant diversity and monthly average precipitation being the most frequently occurring (Table 5).

**Table 4:** Frequency of occurrences of environmental characteristics in selected models describing spider taxonomic, phylogenetic, and functional diversity at a 500 m intermediate scale, as well as the AICc weighted averaged parameter estimates, their standard error, and associated *p* values for each environmental characteristic

Environmental characteristics	Taxonomic diversity (*N* = 1)^A^				Phylogenetic diversity (*N* = 3)^A^					Functional diversity (*N* = 8)^A^			
	*N*^B^	*β*	SE	*p*	*N*^B^	*β*	SE	*p*		*N*^B^	*β*	SE	*p*
*Geomorphologic*													
Aspect													
Slope										2	− 0.04	0.03	0.251
TWI					**3**	− **0.25**	**0.08**	** 0.002**					
Bedrock S0_2_%										2	0.01	0.02	0.714
*Climatic*													
Annual temp										4	0.04	0.05	0.437
Temp. variation													
Monthly avr. Prec.	**1**	− **0.26**	**0.08**	** 0.003**	**3**	− **0.39**	**0.09**	**< 0.001**		5	− 0.05	0.05	0.308
Prec. variation					**3**	** 0.24**	**0.08**	** 0.003**					
*Vegetation*													
Vasc. plant diversity	**1**	** 0.48**	**0.10**	**< 0.001**	**3**	** 0.46**	**0.12**	**< 0.001**		**8**	** 0.12**	**0.05**	**0.019**
Vasc. plant cover	**1**	− **0.32**	**0.13**	** 0.021**	1	0.07	0.15	0.619		6	− 0.09	0.07	0.160
Moss and lichen cover					1	− 0.04	0.07	0.570					
Max vasc. plant height variation													

Bold values reflect statistically significant parameter estimates at an α error of 0.05. The parameter values were averaged across models with a Δ AICc < 2 (i.e., within 2 AICc units of the model with the lowest AICc value) from model sets containing all 4096 possible combinations of environmental characteristics for each diversity dimension

^A^Number of models selected as appropriate based on a Δ AICc < 2

^B^Frequency of occurrences in models selected as appropriate based on a Δ AICc < 2

**Table 5:** Frequency of occurrences of environmental characteristics in selected models describing spider taxonomic, phylogenetic, and functional diversity at a 25 × 25 m local scale, as well as the AICc weighted averaged parameter estimates, their standard error, and associated *p* values for each environmental characteristic

Environmental characteristic	Taxonomic diversity (*N* = 28)^A^				Phylogenetic diversity (*N* = 6)^A^				Functional diversity (*N* = 5)^A^			
	*N*^B^	*β*	SE	*p*	*N*^B^	*β*	SE	*p*	*N*^B^	*β*	SE	*p*
*Geomorphologic*												
Aspect	8	0.01	0.03	0.661	2	0.02	0.05	0.597	4	0.02	0.01	0.122
Slope												
TWI					1	− 0.01	0.03	0.816				
Bedrock S0_2_%	9	− 0.03	0.05	0.595					1	− 3.00 × 10^–**3**^	0.01	0.732
*Climatic*												
Annual temp	7	0.03	0.06	0.645								
Temp. variation	9	− 0.04	0.07	0.603								
Monthly avr. prec.	22	− 0.12	0.10	0.205	**6**	− **0.36**	**0.07**	**< 0.001**	**5**	− **0.07**	**0.01**	**< 0.001**
Prec. variation									1	− 2.00 × 10^–**3**^	0.01	0.801
*Vegetation*												
Vasc. plant diversity	**28**	** 0.21**	**0.05**	**< 0.001**	**6**	** 0.25**	**0.07**	**< 0.001**	**5**	** 0.04**	**0.02**	** 0.021**
Vasc. plant cover	4	− 0.01	0.03	0.807	2	− 0.02	0.05	0.761				
Moss and lichen cover	11	0.02	0.04	0.574					**5**	** 0.03**	**0.01**	** 0.029**
Max vasc. plant height variation	11	0.01	0.02	0.821	1	0.01	0.03	0.793	4	0.03	0.02	0.152

Bold values reflect statistically significant parameter estimates at an α error of 0.05. The parameter values were averaged across models with a Δ AICc < 2 (i.e., within 2 AICc units of the model with the lowest AICc value) from model sets containing all 4096 possible combinations of environmental characteristics for each diversity dimension

^A^Number of models regarded as appropriate based on a Δ AICc < 2

^B^Frequency of occurrences in models selected as appropriate based on a Δ AICc < 2

For spider phylogenetic diversity, three models were selected at the intermediate (Table 4, Supplementary information, Table S4) and six at the local scale (Table 5, Supplementary information, Appendix 1, Table S5). These models contained characteristics from all groups of environmental conditions with topographic wetness index, monthly average precipitation, within year precipitation variation, and vascular plant diversity occurring in all selected models at the intermediate scale (Table 4) and monthly average precipitation and vascular plant diversity being the most frequently occurring at the local scale (Table 5).

For spider functional diversity, eight models were selected at the intermediate (Table 4, Supplementary information, Table S4) and five at the local scale (Table 5, Supplementary information, Appendix 1, Table S5). These models included characteristics from all groups of environmental conditions, with vascular plant diversity being the most frequent characteristics at the intermediate scale (Table 4) and monthly average precipitation, vascular plant diversity, and moss and lichen cover occurring in all selected models at the local scale (Table 5).

### Relative strength of the effects of individual environmental characteristics

Averaged across the selected models, vascular plant diversity had a significant positive effect on spider taxonomic diversity at both the intermediate (*β* = 0.48, SE = 0.10, *p* < 0.001) and the local scale (*β* = 0.21, SE = 0.05, *p* < 0.001). At the intermediate scale, there was also a significant negative effect of monthly average precipitation (*β* = − 0.26, SE = 0.08, *p* = 0.003) and of plant cover (*β* = − 0.32, SE = 0.13, *p* = 0.021).

For spider phylogenetic diversity, there were significant positive effects of vascular plant diversity at both the intermediate (*β* = 0.46, SE = 0.12, *p* < 0.001) and the local scale (*β* = 0.25, SE = 0.07, *p* < 0.001). There were significant negative effects of monthly average precipitation at both the intermediate (*β* = − 0.39, SE = 0.09, *p* < 0.001) and the local scale (*β* = − 0.36, SE = 0.07, *p* < 0.001). At the intermediate scale, there was also a significant positive effect of within year precipitation variation (*β* = 0.24, SE = 0.08, *p* = 0.003) and a significant negative effect of the topographic wetness index (*β* = − 0.25, SE = 0.08, *p* = 0.002).

For spider functional diversity, there were significant positive effects of vascular plant diversity at both the intermediate (*β* = 0.12, SE = 0.05, *p* = 0.019) and the local scale (*β* = 0.04, SE = 0.02, *p* = 0.021). At the local scale, there were also a significant positive effect of moss and lichen cover (*β* = 0.03, SE = 0.01, *p* = 0.029), and a significant negative effect of monthly average precipitation (*β* = − 0.07, SE = 0.01, *p* < 0.001).

## Discussion

The effects of abiotic and biotic environmental conditions on spider diversity differed between the two spatial scales, and also among diversity dimensions. However, the observed scale dependencies in the relative effects of abiotic and biotic environmental conditions did not entirely follow our predictions, i.e., we did not observe stronger abiotic regulation at our coarser scale and stronger effects of biotic conditions at our local scale. Similarly, abiotic conditions, in particular climate characteristics, were important for both phylogenetic and functional diversity and biotic conditions, in particular vascular plant diversity, was important for all diversity dimensions, not only functional diversity.

The observed scale dependencies in the relative importance of environmental conditions for spider diversity agree with previous studies having shown substantial effects of spatial scales for biodiversity regulation (Whittaker et al. 2001; Mirochnitchenko et al. 2021). However, our observation partly contradicts that biotic regulation predominantly takes place at local scales, and that abiotic conditions function as coarse scale filters for regional species pools. Instead, a broader range of environmental conditions appear to have been important for spider diversity at a coarser intermediate scale than at a local scale. This observation is in line with suggestions that it is harder to identify the mechanisms driving diversity over larger areas, such as the longstanding discussions on the driver of the latitudinal patterns of diversity (e.g., Willig et al. 2003). However, our coarser scale covered only 500 m, with approximately the same elevation. Therefore, issues related to processes driving diversity variation across regional or even continental scales may not have been prevalent in our study. Instead, we argue that our results support recent arguments that community assembly is shaped by strong interactions between constrains associated with abiotic conditions and interactions among organisms, and that such interactions cause dynamic processes both across space and time (Kraft et al. 2015; Cadotte and Tucker 2017). Such a conclusion has previously been made for spiders (Müller et al. 2022), and we believe that these studies highlight the need to focus community assembly research more explicitly on the temporal and spatial dynamics in community assembly and maintenance.

Vegetation characteristics had the greatest influence on spider diversity, and vascular plant diversity was the most important vegetation characteristic. This importance of vegetation for spider taxonomic diversity is consistent with previous studies (Uetz 1991; Jiménez-Valverde and Lobo 2007; Bowden and Buddle 2010). In addition to vegetation, climate characteristics, specifically monthly average precipitation, influenced phylogenetic and functional diversity. In the Swedish mountains, vascular plant diversity generally declines at higher elevations (Naud et al. 2019; Måsviken et al. 2020), and there are also strong direct links between plant diversity and local climate, in particular precipitation (Kreft and Jetz 2007). Low temperature and high precipitation are also linked to lower activity of potential prey species (Williams 1961; Antiqueira et al. 2020). We hypothesize that higher precipitation reduces the activity of pray species and that taxonomically richer plant communities offer more complex microhabitats. Since spiders utilize different niches within a vegetation matrix (Schmitz and Suttle 2001), sites with a complex physical structure should permit a broad range of hunting strategies and subsequently also high spider diversity. However, both productivity and vegetation complexity are positively related to the abundance and diversity of prey, which also may increase the spider diversity (e.g., Bowden and Buddle 2010; Yang et al. 2018). Hence, we propose that the observed importance of vegetation and climate is a combination of direct effect that associated the complexity of hunting habitat and indirectly by influencing the diversity and abundance of prey. As both of these environmental characteristics are likely to experience strong shifts with climate change, we suggest that climate alterations may result in dramatic shifts in the spatial distribution of diversity of spiders in high altitude and high latitude environments. However, we recognize that these arguments follow a bottom-up perspective, and that spider communities can also influence vegetation through trophic cascades (Schmitz et al. 2000; Schmitz and Suttle 2001).

We were able to identify a total of 58 spider species, which accounts for almost 8% of the 740 reproducing species in Sweden (Dyntaxa 2022). This proportion of taxa correspond to approximately the proportion (~ 8%) of Sweden’s land area that lies above the tree line (Carlsson et al. 1999). Considering the low productivity of these oroarctic areas, such high taxonomic richness is surprising. However, plants have an even larger proportion of the national species pool present in the Swedish mountain regions (Nilsson 1991; Körner et al. 2017), which we believe could be attributed to large habitat heterogeneity along elevation gradients (Rahbek et al. 2019a, b). These observations exemplify the importance of the mountain areas for the biodiversity of the Scandinavian Peninsula.

Linyphiidae and Lycosidae were the most abundant and taxonomically rich spider families, and they were also the most wide spread across our different sites. These two groups present rather contrasting ecological characteristics, with Linyphiidae being primarily small web building spiders and Lycosidae being ground dwelling active hunters. Måsviken et al. (2023) showed that there are both elevational and geographic variation in the relative composition of spider communities at our study sites, with locations at high elevations primarily being dominated by small Linyphiidae spiders and the actively hunting Lycosidae primarily occurring at lower elevations. Previous studies have made similar observations (Entling et al. 2010), and small arthropod species have been regarded as better adapted to areas with low productivity partly due to short growing seasons and low prey availability (Høye and Hammel 2010; Ameline et al. 2018). In addition, it is likely that dispersal strategies also have influenced the spatial variation in spider diversity. We believe that such variation exemplifies how evolutionary processes, the phenotypes these processes have resulted in, and the abiotic environment interact in shaping the composition of local communities, and hence also spatial variation in biodiversity.

While we generally regard our results to be robust, we provide some caveats to or study. First, while sampling method may affect the diversity obtained from spider surveys (Churchill and Arthur 1999; Ernst et al. 2016), we exclusively used pitfall traps for spider sampling. However, we recorded a wide range of both taxonomically and functionally different spiders, suggesting that our sampling strategy did not constrain the collected spiders to specific taxonomic or functional groups. Second, we created our phylogeny using a single genetic marker in the COI mitochondrial region. Thus, a phylogeny using multiple markers, or even complete genome sequences, may have generated more informed phylogenetic relationships (Macías-Hernández et al. 2020). However, as the COI region is the most widely used barcode region for arthropods (Coddington et al. 2016; Blagoev et al. 2016; Andújar et al. 2018; Liu et al. 2020), we regard it to provide a more robust hypothesis of phylogenetic relationships than markers with more informative genetic sequences but poorer taxonomic cover (Nixon 2001). Third, an obvious shortcoming with our study is the lack of species level trait data for spiders. However, while we recognize that this lack of species-specific trait data may have caused us to underestimate functional diversity, many of the selected traits are phylogenetically conservative. We, therefore, believe that such underestimations were not severe. The recent creation of a more comprehensive spider trait database, World spider trait database (Pekár et al. 2021), is commendable but the taxonomic cover is yet low and we encourage additional studies reporting species level data on ecological traits of spiders. Fourth, we used predictors with various spatial resolution for our analyses, with the resolution for our precipitation data being substantially coarser (approx. 1 km^2^) than our finest sampling unit (i.e., 25 × 25 m). Although contrasting spatial scales may influence spatial analyses (Connor et al. 2018), we regard the coarse scale of our climate data to be acceptable since climate and, in particular, precipitation generally does not vary largely within small spatial scales. Finally, we choose to use tree-based metrics of phylogenetic and functional diversity. Although such metrics are constrained to not allow for negative relationship between taxonomic richness and phylogenetic or functional diversity, the strength of the effect of taxonomic richness is heavily dependent on how similar species are in their evolutionary history and phenotypic characteristics (e.g., Dalerum et al. 2012). We regard this as a desirable property. Furthermore, we do not regard it appropriate to select an index that would allow for a decline in diversity with the addition of taxa, or conversely, that the deletion of taxa could lead to an increased diversity (Petchey and Gaston 2007).

To conclude, we observed variation in the relative effects of abiotic and biotic conditions for spider diversity both across spatial scales and among taxonomic, phylogenetic, and functional diversity dimensions. However, this variation did not fully conform to our predictions, i.e., that abiotic conditions primarily would be important at coarse spatial scales and for phylogenetic diversity, and that biotic conditions primarily would be important at local scales and for functional diversity. Instead, we believe that our results indicate that community assembly is shaped by interactions between abiotic constrains in species distributions and biotic conditions. Environmental conditions associated with vegetation and precipitation were the most important for spider diversity across diversity dimensions and spatial scales, which we attribute to direct effects on diversity through shifting habitat heterogeneity and indirect effects linked to the diversity and abundance of prey. Since both of these environmental conditions are likely to see dramatic changes with an altered climate, we believe that there may be substantial alterations to spider diversity in the near future. We argue that there is a need to focus research on biodiversity regulation on how abiotic regulation of species ranges influences the species interactions within ecological communities, and in particular evaluating how such effects shift among spatial scales and ecological contexts.

## Supplementary Information

Below is the link to the electronic supplementary material.

Supplementary file1 (PDF 516 KB)

## Data Availability

Data supporting the results is available on figshare (https://figshare.com/10.6084/m9.figshare.22643935).
