# Supplementary material for: The relative importance of abiotic and biotic environmental conditions for taxonomic, phylogenetic, and functional diversity of spiders across spatial scales
Source: Oecologia. 2023 Jun 1;202(2):261–73. doi: 10.1007/s00442-023-05383-0 (PMC10307692; doi:10.1007/s00442-023-05383-0)
Supplement: Supplementary file 1 — Supplementary file1 (PDF 516 KB) [file 442_2023_5383_MOESM1_ESM.pdf]

SUPPORTING INFORMATION

The relative importance of abiotic and biotic environmental conditions for taxonomic, phylogenetic and functional diversity of spiders across spatial scales

Johannes Måsviken[123] Love Dalén[123] Karin Norén[1] Fredrik Dalerum[145]

[1]Department of Zoology, Stockholm University, Stockholm, Sweden

[2]Department of Bioinformatics & Genetics, Swedish Museum of Natural History, Stockholm, Sweden

[3] Centre for Palaeogenetics, Stockholm

[4] Biodiversity Research Institute (University of Oviedo-Principality of Asturias-CSIC), Spanish National Research Council, Research Building, Mieres Campus, 33600 Mieres, Spain

[5] Mammal Research Institute, Department of Zoology and Entomology, University of Pretoria, Hatfield, South Africa

Appendix 1, Supplementary figures and tables

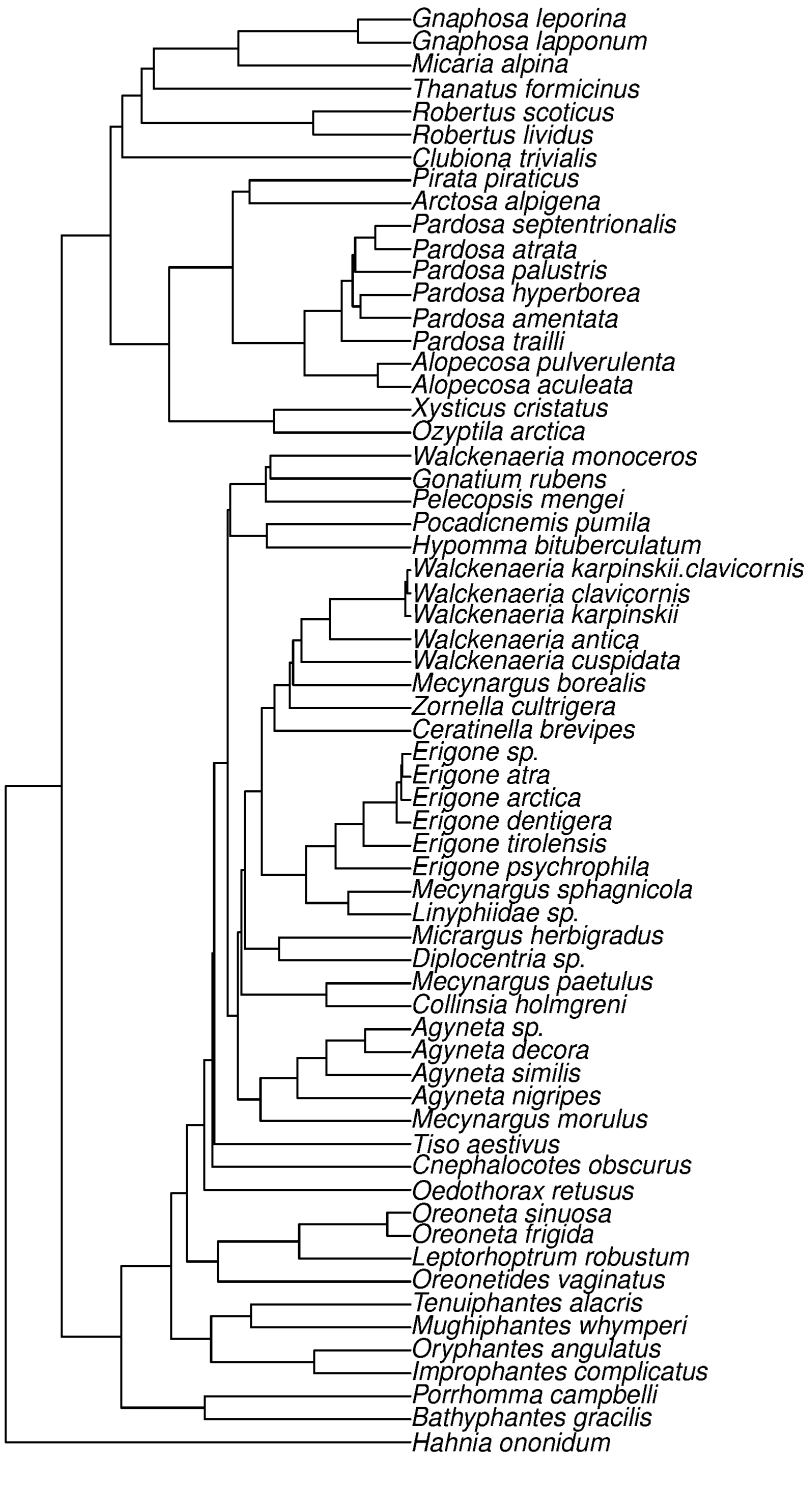

Figure S1. Phylogeny used to quantify phylogenetic diversity of spider assemblages. The phylogeny was based on genetic sequences from the mitochondrial COI region.

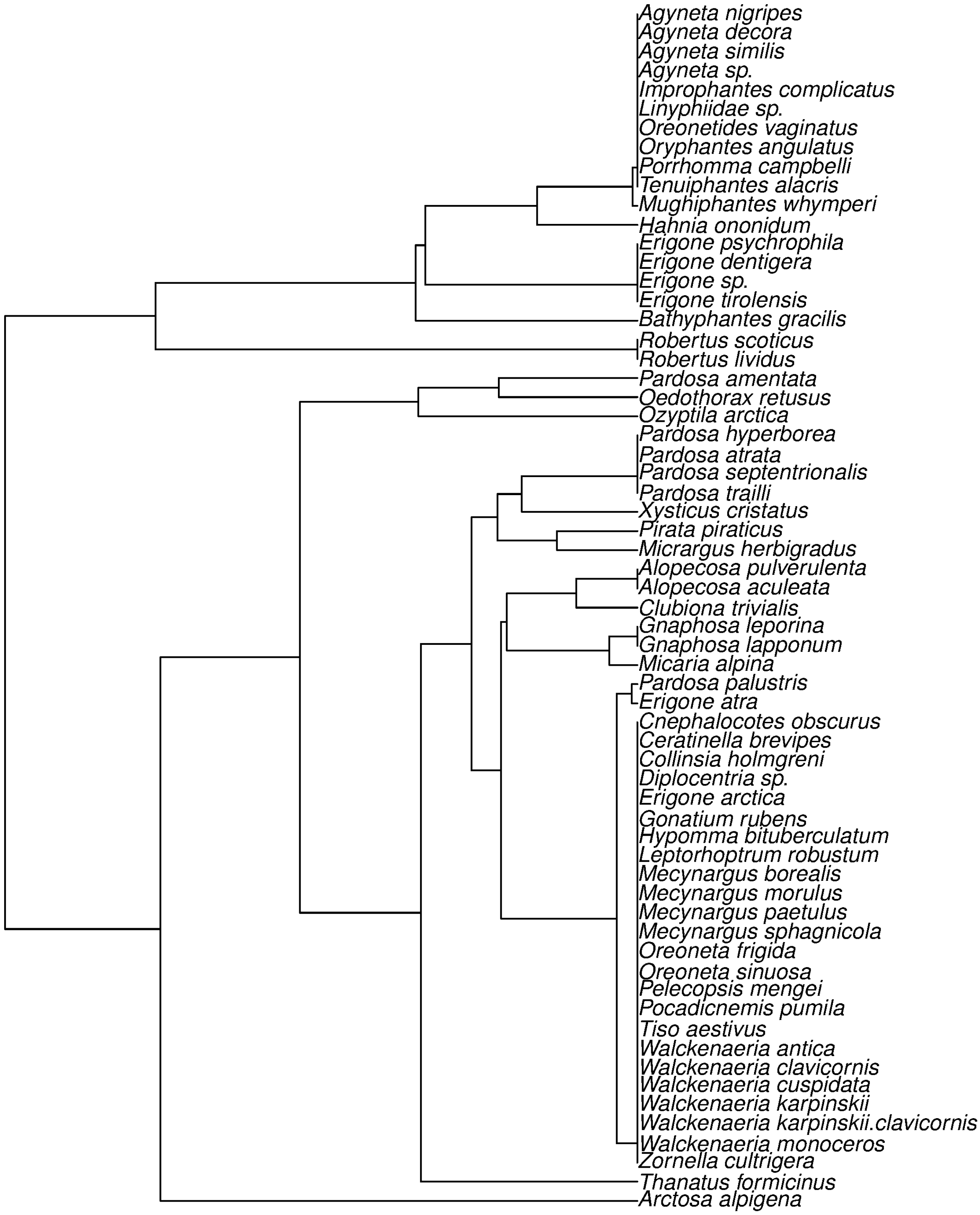

Figure S2. Functional dendrogram of spiders used to quantify functional diversity of spider assemblages. The dendrogram was based on a trait matrix consisting of six traits related to size, hunting behaviour, diet preferences and dispersal abilities.

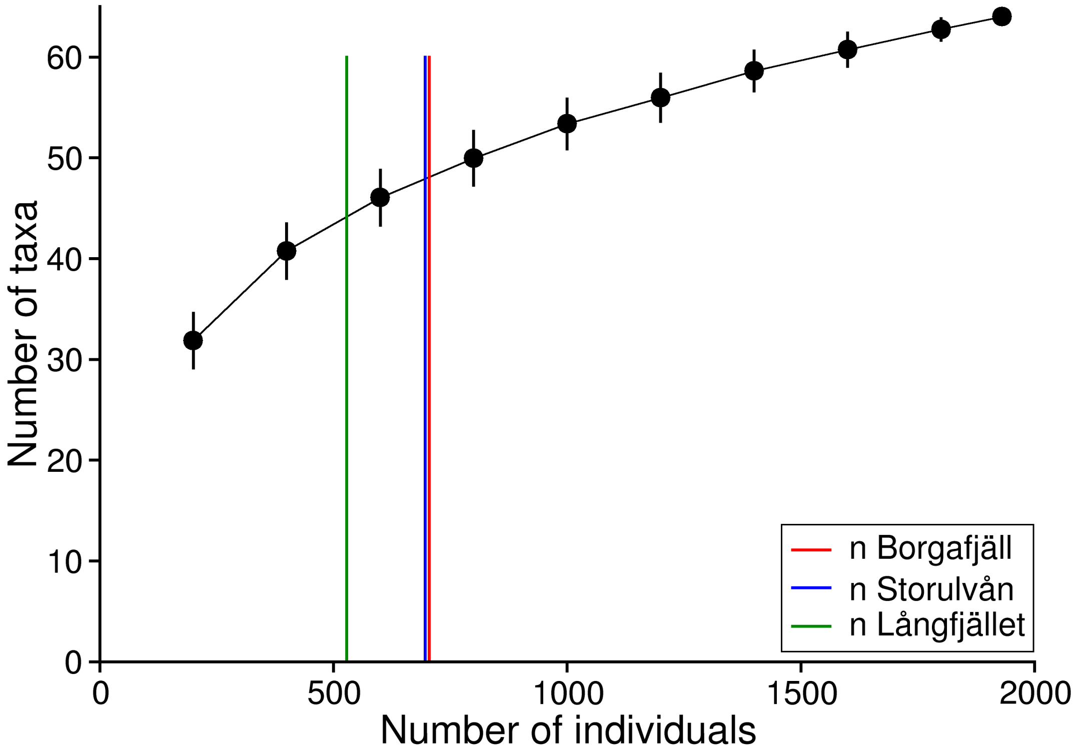

Figure S3. Species accumulation curve indicating the average number of taxa identified from 1000 randomly samples of sizes ranging from 50 to 1930 individual spiders, including indications for the sample sizes at each of the three sites Borgafjäll, Storulvån and Fulufjället.

Table S1. Characters used for the spider trait matrix, their data type, and the taxonomic accuracy, quantified as the percentage of taxa for which we had trait information at the taxonomic rank of species, genus, subfamily, family, and nearest family.

Character	Type & levels	Species	Genus	Subfamily	Family	Nearest family
Dispersal	Factor<br>Frequent<br>Occasionally	32 %	41 %	0 %	27 %	0 %
Hunting mode	Factor<br>Active<br>Web<br>Multiple	32 %	41 %	0 %	27 %	0 %
Web type	Factor<br>No web<br>Sheet-space	21 %	46 %	31 %	2 %	0 %
Main prey	Factor<br>Coleoptera<br>Collembola<br>Diptera<br>Hemiptera<br>Hymenoptera	10 %	18 %	56 %	11 %	6 %
Diet index	Continuous	8 %	11 %	49 %	25 %	5 %
Average body length	Continuous	92 %	6 %	0 %	2 %	0 %

Table S2. Moran´s statistics, evaluating spatial autocorrelation in the residuals, for all models containing the full set of environmental characteristics.

**Model**	**Moran I**	**Z**	**P**
**Intermediate**			
Taxonomic diversity	-0.07	-0.68	0.751
Phylogenetic diversity	-0.09	-0.99	0.840
Functional diversity	-0.10	-1.17	0.878
**Local**			
Taxonomic diversity	-0.03	-1.00	0.839
Phylogenetic diversity	-0.02	-0.62	0.734
Functional diversity	-0.02	-0.82	0.794

Table S3. The number of sampled individuals of each spider taxon at the different sites (BF-Borgafjäll, SU-Storulvån, LF-Långfjället) and the two different spatial scales, intermediate and local.

	**Intermediate**			**Local**		
**Taxa**	**BF**	**SU**	**LF**	**BF**	**SU**	**LF**
Agyneta decora	6	4	0	6	4	0
Agyneta nigripes	32	8	1	32	13	2
Agyneta similis	4	5	5	4	5	6
Agyneta sp.	0	0	2	0	0	2
Alopecosa aculeata	13	3	3	13	5	3
Alopecosa pulverulenta	0	0	1	0	0	1
Arctosa alpigena	9	10	1	9	10	1
Bathyphantes gracilis	4	0	0	4	1	0
Ceratinella brevipes	0	2	0	0	2	0
Clubiona trivialis	0	0	0	0	0	1
Cnephalocotes obscurus	0	1	0	0	1	0
Collinsia holmgreni	65	129	0	65	161	0
Diplocentria sp.	1	0	0	1	0	0
Erigone arctica	0	44	0	0	76	0
Erigone atra	3	9	6	3	21	6
Erigone dentigera	0	1	0	0	1	0
Erigone psychrophila	2	16	1	2	16	1
Erigone sp.	1	4	0	1	4	0
Erigone tirolensis	171	59	0	171	78	0
Gnaphosa lapponum	0	7	14	0	7	16
Gnaphosa leporina	15	23	35	15	25	46
Gonatium rubens	4	0	2	4	0	3
Hahnia ononidum	0	2	1	0	2	1
Hypomma bituberculatum	2	1	1	2	1	2
Improphantes complicatus	0	2	0	0	2	0
Leptorhoptrum robustum	0	1	0	0	4	0
Linyphiidae sp.	1	8	0	1	8	0
Mecynargus borealis	1	0	0	1	0	0
Mecynargus morulus	0	0	1	0	1	1
Mecynargus paetulus	22	0	0	22	0	0
Mecynargus sphagnicola	0	0	1	0	0	1
Micaria alpina	1	4	5	1	6	5
Micrargus herbigradus	0	0	1	0	0	1
Mughiphantes whymperi	0	2	0	0	2	0

(continued)	**Intermediate**			**Local**		
Oedothorax retusus	10	3	0	10	4	0
Oreoneta frigida	0	0	0	0	1	0
Oreoneta sinuosa	3	5	1	3	5	1
Oreonetides vaginatus	3	4	1	3	4	1
Oryphantes angulatus	0	1	0	0	1	0
Ozyptila arctica	1	6	10	1	6	13
Pardosa amentata	70	11	2	70	35	2
Pardosa atrata	0	1	5	0	2	5
Pardosa hyperborea	4	48	25	4	49	46
Pardosa palustris	217	58	230	217	74	289
Pardosa septentrionalis	0	0	0	0	1	0
Pardosa trailli	0	0	19	0	0	19
Pelecopsis mengei	1	13	6	1	13	8
Pirata piraticus	3	0	0	3	0	0
Pocadicnemis pumila	0	0	0	0	0	2
Porrhomma campbelli	0	0	0	0	1	0
Robertus lividus	1	0	0	1	0	0
Robertus scoticus	0	0	1	0	0	1
Tenuiphantes alacris	1	0	0	1	0	0
Thanatus formicinus	0	0	1	0	0	1
Tiso aestivus	8	20	23	8	28	27
Walckenaeria antica	1	0	0	1	0	0
Walckenaeria clavicornis	7	0	0	7	0	0
Walckenaeria cuspidata	1	2	2	1	2	2
Walckenaeria karpinskii	0	9	0	0	11	1
Walckenaeria karpinskii/clavicornis	16	0	0	16	0	0
Walckenaeria monoceros	0	0	1	0	0	1
Xysticus cristatus	1	0	8	1	0	11
Zornella cultrigera	0	3	0	0	3	0
Total no species	36	37	32	36	42	35
Total no spiders	705	529	416	705	696	529

Table S4. Environmental characteristics included in models selected as appropriate for the intermediate scale from model sets containing all possible combinations of environmental predictors, their AICc scores relative to the model with the lowest AICc value (Δ AICc), as well as their Akaike weight (W AICc). Models were regarded as appropriate if they had Δ AICc values less than 2. The models were selected from a full set of 4096 candidate models for each diversity dimension.

Predictors	Δ AICc	W AICc
**Taxonomic diversity**		
Monthly avr. prec. + Vasc. plant diversity + Vasc. plant cover	0	1.00
**Phylogenetic diversity**		
Monthly avr. prec. + Prec. variation + Vasc. plant diversity + TWI	0	0.40
Monthly avr. prec. + Prec. variation + Moss & lichen cover + Vasc. plant diversity + TWI	0.37	0.33
Monthly avr. prec. + Prec. variation + Vasc. plant diversity + TWI + Vasc. plant cover	0.71	0.28
**Functional diversity**		
Annual temp. + Vasc. plant diversity + Slope + Vasc. plant cover	0	0.20
Monthly avr. prec. + Vasc. plant diversity + Vasc. plant cover	0.29	0.17
Monthly avr. prec. + Vasc. plant diversity + Slope + Vasc. plant cover	0.35	0.17
Monthly avr. prec. + Vasc. plant diversity	1.05	0.12
Annual temp. + Bedrock S02% + Vasc. plant diversity + Slope + Vasc. plant cover	1.41	0.10
Monthly avr. prec. + Bedrock S02% + Vasc. plant diversity + Slope + Vasc. plant cover	1.76	0.08
Annual temp. + Vasc. plant diversity + Slope	1.81	0.08
Monthly avr. prec. + Annual temp. + Vasc. plant diversity + Slope + Vasc. plant cover	1.87	0.08

Table S5. Environmental characteristics included in models selected as appropriate for the local scale from model sets containing all possible combinations of environmental predictors, their AICc scores relative to the model with the lowest AICc value (Δ AICc), as well as their Akaike weight (W AICc). Models were regarded as appropriate if they had Δ AICc values less than 2.

Predictors	Δ AICc	W AICc
**Taxonomic diversity**		
Monthly avr. prec. + Vasc. plant diversity	0	0.06
Monthly avr. prec. + Moss & lichen cover + Vasc. plant diversity	0.02	0.06
Annual temp. + Vasc. plant diversity	0.13	0.06
Monthly avr. prec. + Bedrock S02% + Moss & lichen cover + Vasc. plant diversity	0.43	0.05
Monthly avr. prec. + Bedrock S02% + Vasc. plant diversity + Temp. variation	0.44	0.05
Monthly avr. prec. + Aspect + Bedrock S02% + Vasc. plant diversity + Temp. variation	0.49	0.05
Monthly avr. prec. + Vasc. plant diversity + Temp. variation	0.60	0.05
Monthly avr. prec. + Bedrock S02% + Vasc. plant diversity	0.76	0.04
Monthly avr. prec. + Aspect + Vasc. plant diversity + Temp. variation	0.92	0.04
Monthly avr. prec. + Vasc. plant diversity + Vasc. plant cover	1.07	0.04
Monthly avr. prec. + Aspect + Moss & lichen cover + Vasc. plant diversity	1.15	0.03
Annual temp. + Moss & lichen cover + Vasc. plant diversity	1.16	0.03
Annual temp. + Aspect + Vasc. plant diversity	1.21	0.03
Monthly avr. prec. + Aspect + Vasc. plant diversity	1.22	0.03
Monthly avr. prec. + Bedrock S02% + Moss & lichen cover + Vasc. plant diversity + Temp. variation	1.22	0.03
Annual temp. + Bedrock S02% + Vasc. plant diversity	1.22	0.03
Annual temp. + Vasc. plant diversity + Vasc. plant cover	1.29	0.03
Monthly avr. prec. + Vasc. plant diversity + Max height variation	1.35	0.03
Monthly avr. prec. + Moss & lichen cover + Vasc. plant diversity + Temp. variation	1.45	0.03
Monthly avr. prec. + Aspect + Bedrock S02% + Moss & lichen cover + Vasc. plant diversity + Temp. variation	1.46	0.03
Monthly avr. prec. + Moss & lichen cover + Vasc. plant diversity + Max height variation	1.47	0.03
Monthly avr. prec. + Aspect + Bedrock S02% + Moss & lichen cover + Vasc. plant diversity	1.62	0.03
Annual temp. + Vasc. plant diversity + Max height variation	1.82	0.02
Monthly avr. prec. + Vasc. plant diversity + Max vasc. plant height variation + Temp. variation	1.83	0.02
Monthly avr. prec. + Bedrock S02% + Vasc. plant diversity + Vasc. plant cover	1.85	0.02
Monthly avr. prec. + Annual temp. + Vasc. plant diversity	1.87	0.02
Monthly avr. prec. + Moss & lichen cover + Vasc. plant diversity + Vasc. plant cover	1.89	0.02
Monthly avr. prec. + Aspect + Moss & lichen cover + Vasc. plant diversity + Temp. variation	1.92	0.02

(continued)<br>**Predictors**	**Δ AICc**	**W AICc**
**Phylogenetic diversity**		
Monthly avr. prec. + Vasc. plant diversity	0	0.28
Monthly avr. prec. + Aspect + Vasc. plant diversity	0.40	0.23
Monthly avr. prec. + Vasc. plant diversity + Max height variation	1.39	0.14
Monthly avr. prec. + Vasc. plant diversity + TWI	1.60	0.13
Monthly avr. prec. + Vasc. plant diversity + Vasc. plant cover	1.70	0.12
Monthly avr. prec. + Aspect + Vasc. plant diversity + Vasc. plant cover	2.00	0.10
**Functional diversity**		
Monthly avr. prec. + Aspect + Moss & lichen cover + Vasc. plant diversity + Max height variation	0	0.36
Monthly avr. prec. + Aspect + Bedrock S02% + Moss & lichen cover + Vasc. plant diversity + Max height variation	1.23	0.20
Monthly avr. prec. + Aspect + Moss & lichen cover + Vasc. plant diversity	1.74	0.15
Monthly avr. prec. + Aspect + Prec. variation + Moss & lichen cover + Vasc. plant diversity + Max height variation	1.75	0.15
Monthly avr. prec. + Moss & lichen cover + Vasc. plant diversity + Max height variation	1.98	0.14

Appendix 2, Supplementary methods

Sequence alignment and tree construction

We obtained sequences from GenBank (Sayers et al. 2021) in March of 2021 for all but eleven of the collected spider taxa (*Agyneta similis, Diplocentria sp., Gnaphosa lapponum, Gnaphosa leporina, Mecynargus morulus, Mugiphantes whymperi, Oreoneta frigida, Oreoneta sinuosa, Oryphantes angulatus, Porrhomma campbelli, Zornella cultrigera*), which lacked available sequences. Observe that the taxonomy of the genus *Oreoneta* is poorly investigated. For these eleven taxa, we generated sequences by extracting DNA from our own morphologically identified specimens. The DNA extraction is described in the section below. If we obtained multiple sequences, we first aligned them for each taxon separately to generate a consensus sequence to be used in the full alignment. Likewise, sequences for specimens identified to a higher taxonomic rank or belonging to one of two closely related species (*Walckenaeria karpinskii/clavicornis*) were obtained by first aligning all sequences from our identified species within that taxonomic rank (family/genus etc.) to one consensus sequence before the full alignment. All sequences were aligned using the software Geneious Prime (version 2021.1.1., Biomatters Ltd., Auckland, New Zealand) using a global alignment with free end gaps and a 65 % similarity cost matrix, gap open penalty “12”, gap extension penalty “3” and two refinement iterations. This alignment method uses an iterative approach of pairwise alignments, described by Feng and Doolittle (1987).

A phylogenetic tree was built using a maximum likelihood approach by running the alignment of DNA sequences in the software IQ Tree (Nguyen et al. 2015) with a combination of ultrafast bootstrap (Hoang et al. 2018) and SH-aLRT branch test (Guindon et al. 2010), both with 1000 iterations. Additionally, the IQ Tree software were let to automatically choose the best fitted substitution model for our data based on BIC (Bayesian Information Criterion).

DNA extraction

For taxa lacking publicly available COI sequences, we generated them using the following methods. DNA extraction was done by removing a couple of legs, depending on the size of the spider. If several specimens were available, one spider from each site was used as replicates. The spider legs were crushed in a tube to increase DNA yield prior to the addition of a lysis buffer containing proteinase K (either Thermo Scientific Cell and Tissue Kit lysis buffer or buffer from Marquina (2022)), after which the sample was incubated under rotation at 56ºC for 2.5 hours or overnight. After incubation the lysate was transferred to a plate and extracted using Thermo Scientific Kingfisher Flex extraction robot, following standard protocol with reagents from the Thermo Scientific Cell and Tissue Kit. The DNA extract was amplified using the COI primers BF3 and BR2 (Elbrecht and Leese 2017; Elbrecht et al. 2019) and using a PCR mix consisting of one Illustra Hot Start Mix RTG bead (GE Healthcare Life Sciences, Freiburg, Germany), 21µl lab grade water, 2µl DNA-extract, 1µl forward and 1µl reverse primer. The PCR were run using the following temperature protocol: initial Taq-activation and denaturalization phase, 95ºC for 5 minutes, followed by 30 cycles of 95ºC for 30 seconds (s), annealing phase at 48ºC for 45s followed by an extension phase of 68ºC for 45s and the program was ended by a final extension phase of 72ºC for 10 minutes. The PCR products were then checked on an agarose gel for amplification success and amplicon length. Negative controls were used throughout the lab work from lysis, extraction, and PCR but were discarded after confirming that they lacked sign of amplification. PCR

products were cleaned using ExoSAP-IT (Bell 2008) and sent to Macrogen Inc (Amsterdam, the Netherlands) for sanger sequencing of both forward and reverse complimentary sequences.

Calculations of environmental characteristics

We extracted geomorphological and climate characteristics using 25x25 m square polygons centred on the central coordinate of each sample station. For the intermediate scale, we used the three sampling stations pooled whereas we used individual values for each station for the local scale. These characteristics were calculated per pixel and represented as averages across a total area of 1875 m2 for each transect (intermediate scale) and 625 m2 for each station (local scale). The geomorphological characteristics aspect, slope and TWI were calculated from a digital elevation model (DEM) with a pixel size of 2 m from the Swedish land survey (Lantmäteriet 2022). Aspect and slope were expressed in degrees and calculated following Horn's algorithm (1981) using a 3x3 pixel neighbourhood size. For aspect, the values indicate the azimuth of the slope with 0 degrees representing the geographic north, going clockwise. TWI describes the water accumulation potential across a landscape and was calculated as the natural logarithm of the ratio of an upslope catchment area and the tangent of the slope of the target pixel (Beven and Kirkby 1979). The catchment area was calculated using the multiple flow direction algorithm (Quinn et al. 1991), which has been suggested as appropriate for ecological studies (Kopecký and Čížková 2010). Bedrock silica content was based on bedrock information from the geological survey of Sweden (SGU 2021) and quantified as a factor with four levels based on standard classification of the percentage silica (SiO2), felsic (>65 %), intermediate (52-65 %, set to the average 58.5 %), mafic (45-52 %, set to the average 48.5 %) and ultramafic (<45 %).

Annual temperature and monthly temperature variation values were obtained from a temperature model with a 50 m resolution developed by Meineri and Hylander (2017). Values for monthly average precipitation and within year precipitation variation were obtained from WorldClim 2.1 data with a resolution of 30 arc seconds (~1 km2) as averages for the period 1970-2000 (Fick and Hijmans 2017). GIS analysis was done using QGIS Desktop (version 3.10.14, https://www.qgis.org).

We derived vegetation characteristics from our own surveys of 1 m2 sample plots placed at the same location as the pitfall traps (Figure 1e). Within each of the sampling plots all vascular plant taxa were recorded to species or closest possible taxonomical rank, following the taxonomy by the database Dyntaxa (2021). The cover of alive vascular plants, mosses, lichens, and dead plant tissue was visually estimated to the nearest half percent using an 10x10cm intercept grid in the sampling plots. Lichen cover was defined as percentage of plot covered by non-crustaceous lichens. We estimated vascular plant diversity using the Shannon index (Shannon 1948), based on the relative abundance of each species in each plot. Relative abundances were estimated using an intercept method modified from the International Tundra Experiment (Naud et al. 2019). We inserted one intercept stick at 25 randomly placed points along the 10x10 cm grid in every plot. The same 25 points within the grid were kept for all plots. In addition, we measured the height of each plant touching the stick at the 25 intercept points. These measurements were used to calculate the variation in vascular plant height and providing information on the physical vegetation structure.

References

Beven KJ, Kirkby MJ (1979) A physically based, variable contributing area model of basin hydrology. Hydrol Sci Bull 24:43–69. https://doi.org/10.1080/02626667909491834

Bell JR (2008) A simple way to treat PCR products prior to sequencing using ExoSAP-IT®. Biotechniques 44:834. https://doi.org/10.2144/000112890

Dyntaxa (2022) Swedish taxonomic database. https://namnochslaktskap.artfakta.se/

Elbrecht V, Braukmann TWA, Ivanova N V., et al (2019) Validation of COI metabarcoding primers for terrestrial arthropods. PeerJ 7:e7745. https://doi.org/10.7717/PEERJ.7745

Elbrecht V, Leese F (2017) Validation and development of COI metabarcoding primers for freshwater macroinvertebrate bioassessment. Front Environ Sci 5:11. https://doi.org/10.3389/fenvs.2017.00011

Feng D-F, Doolittle RF (1987) Progressive sequence alignment as a prerequisite to correct phylogenetic trees. J Mol Evol 25:351–360. https://doi.org/10.1007/BF02603120

Fick SE, Hijmans RJ (2017) WorldClim 2: new 1-km spatial resolution climate surfaces for global land areas. Int J Climatol 37:4302–4315. https://doi.org/10.1002/joc.5086

Guindon S, Dufayard J-F, Lefort V, et al (2010) New algorithms and methods to estimate maximum-likelihood phylogenies: Assessing the performance of PhyML 3.0. Syst Biol 59:307–321. https://doi.org/10.1093/sysbio/syq010

Hoang DT, Chernomor O, Von Haeseler A, et al (2018) UFBoot2: Improving the ultrafast bootstrap approximation. Mol Biol Evol 35:518–522. https://doi.org/10.1093/molbev/msx281

Horn BKP (1981) Hill shading and the reflectance map. Proc IEEE 69:14–47. https://doi.org/10.1109/PROC.1981.11918

Kopecký M, Čížková Š (2010) Using topographic wetness index in vegetation ecology: Does the algorithm matter? Appl Veg Sci 13:450–459. https://doi.org/10.1111/j.1654-109X.2010.01083.x

Lantmäteriet (2022) Geo data. GSD-Höjddata grid 2+ (DEM 2 m) GDS-Ortofoto RGB and CIR. Lantmäteriet, Gävle

Marquina D, Roslin T, Łukasik P, Ronquist F (2022) Evaluation of non-destructive DNA extraction protocols for insect metabarcoding: gentler and shorter is better. Metabarcoding and Metagenomics 6:e78871. https://doi.org/10.3897/mbmg.6.78871

Meineri E, Hylander K (2017) Fine-grain, large-domain climate models based on climate station and comprehensive topographic information improve microrefugia detection. Ecography 40:1003–1013. https://doi.org/10.1111/ecog.02494

Naud L, Måsviken J, Freire S, et al (2019) Altitude effects on spatial components of vascular plant diversity in a subarctic mountain tundra. Ecol Evol 9:4783–4795. https://doi.org/10.1002/ECE3.5081

Nguyen LT, Schmidt HA, Von Haeseler A, Minh BQ (2015) IQ-TREE: A fast and effective stochastic algorithm for estimating maximum-likelihood phylogenies. Mol Biol Evol 32:268–274. https://doi.org/10.1093/molbev/msu300

Sayers EW, Cavanaugh M, Clark K, et al (2021) GenBank. Nucleic Acids Res 49:D92–D96. https://doi.org/10.1093/nar/gkaa1023

SGU (2021) Digital bedrock map 1:50 000-1:250 000, Swedish geological survey, Uppsala

Shannon CE (1948) A mathematical theory of communication. Bell Syst Tech J 27:379–423. https://doi.org/10.1002/j.1538-7305.1948.tb01338.x

Quinn P, Beven K, Chevallier P, Planchon O (1991) The prediction of hillslope flow paths for distributed hydrological modelling using digital terrain models. Hydrol Process 5:59–79. https://doi.org/10.1002/hyp.3360050106
